# Molecular Docking, Pharmacophore, and 3D-QSAR Approach: Can Adenine Derivatives Exhibit Significant Inhibitor Towards Ebola Virus?

**DOI:** 10.2174/1874104501711010127

**Published:** 2017-11-30

**Authors:** Amit Rai, Mohamed H. Aboumanei, Suraj P. Verma, Sachidanand Kumar, Vinit Raj

**Affiliations:** 1 Department of Pharmaceutical Sciences, Babasaheb Bhimrao Ambedkar University, Vidya Vihar, Raebareli Road, Lucknow 226025, India; 2 Egyptian Atomic Energy Authority, Cairo 11371, Egypt; 3 Department of Pharmaceutical Sciences, Kumaun University, Bhimtal Campus, Nainital Uttarakhand, India; 4 Quintiles IMS, II Floor, Etamin Block, Prestige Technology Park II, Sarjapur-Marathahalli Outer Ring Road, Bangalore – 560103, India

**Keywords:** Ebola virus, Pharmacophore, 3D-QSAR, Homological modeling and Molecular docking, Pharmacophore models

## Abstract

**Introduction:**

Ebola Virus Disease (EVD) is caused by Ebola virus, which is often accompanied by fatal hemorrhagic fever upon infection in humans. This virus has caused the majority of deaths in human. There are no proper vaccinations and medications available for EVD. It is pivoting the attraction of scientist to develop the potent vaccination or novel lead to inhibit Ebola virus.

**Methods & Materials:**

In the present study, we developed 3D-QSAR and the pharmacophoric model from the previous reported potent compounds for the Ebola virus.

**Results & Discussion:**

Results & Discussion: The pharmacophoric model AAAP.116 was generated with better survival value and selectivity. Moreover, the 3D-QSAR model also showed the best r2 value 0.99 using PLS factor. Thereby, we found the higher F value, which demonstrated the statistical significance of both the models. Furthermore, homological modeling and molecular docking study were performed to analyze the affinity of the potent lead. This showed the best binding energy and bond formation with targeted protein.

**Conclusion:**

Finally, all the results of this study concluded that 3D-QSAR and Pharmacophore models may be helpful to search potent lead for EVD treatment in future.

## 1 INTRODUCTION

Ebola infection disease (EVD) is a zoonosis caused by infection due to filoviruses of the genus Ebola virus. The EVD is more prevalent in Africa except other countries. There are mostly found in species such as Sudan ebolavirus, Zaire ebolavirus, Budibugyo ebolavirus and Tai Forest ebolavirus [1]. These viruses cause regularly lethal hemorrhagic fever in people [1]. This infection’s transmission from wildlife has generally been connected to individuals taking care of wild animals for bushmeat [2]. Even, natural reservoir host of Ebola virus remains unknown. Many researchers believed that the virus is animal-borne and that bats are the majority likely reservoir (Author: This highlighted phrase seems vague and must be re-phrased). It is generally vital to see how supply components, together with environmental conditions and human conduct, add to Ebola infection flare-ups [3]. Lately, biogeographically investigations have highlighted the significance of potential stores (animals that can harbor the 4 pathogens inconclusively with no evil impacts) in clarifying the spatial collection of human irresistible sicknesses overall [4]. Biogeography has contributed extensively to inquiries of irresistible disease biology, administration and reconnaissance [5]. Albeit likely supply species for the Ebola infection have been highlighted by a few creators [6], existing models depicting the appropriation of the infection have either not considered the commitment of supplies in managing its nearness [7], or have accepted that lone few species, suspected to be the repositories for the infection, are important in the biogeography of the infection [8]. Along these lines, forcing limitations to the determination of animal species considered in a dispersion model may under speak to the zoological substrate that could decide the circulation of the infection. Indeed, the part of specific bat species as genuine repositories of Ebola infection is still under discourse, and it has been practically proved that there is a critical infection overflow among vertebrate species not suspected to be the supplies [9]. Ebola infection, in light of factors characterizing the current sorts of mammalian conveyances in Africa, ought to better depict the infection events recorded in untamed life than a model in view of natural descriptors alone. The known writing with respect to occasions of Ebola infection rise, either EVD episodes or recorded nearness of the infection in non-human warm blooded animals(Author: This highlighted phrase seems vague and must be re-phrased).

The previous literature regarding the adenine-sugar containing derivatives has the potent inhibitor for Ebola Virus [10]. The aim of present study was to focus on providing the essential atomic pharmacophore features for the development of potent lead as an Ebola virus inhibitor. Another approach, 3D-QSAR provided essential atomic substitution factor, which was responsible for increasing activity profile towards the target and molecular docking also provided active site information of the target.

This above study parameter revealed and provided the potent model for the development of novel compounds for the Ebola virus. However, this study would be a milestone in the path, who is working for the search of the lead as a potent Ebola virus inhibitor.

## 2 MATERIAL AND METHODS

### 2.1 Design and Database

In the present study, we have searched literature on Ebola virus inhibitor, where we observed that a few data is available on the inhibitor of Ebola virus. Thereby, we used the series of 9 potent compounds containing adenine derivatives shown in Fig. (**1**). The chemical structure was drawn using by ChemDraw 12.0 and their geometry was optimized with the Gauss View 5.0. software. On the other hand, the energy minimization was evaluated by the ChemPro3D. Finally, bond length and Angle of atoms were optimized *via* Argus Lab. (http://autodock.scripps.edu/) along with its Lamarckian Genetic Algorithm (LGA).

Adenine derivatives were used 50% inhibition concentration (IC_50_) towards Ebola virus. In the present study, The IC_50_ was used for the pharmacophore generation and QSAR analysis. Further, the IC_50_converted into the corresponding pIC_50_ [−log(IC_50_)] and used as dependent variables QSAR and pharmacophore calculations (Table **1**).

### 2.2 Pharmacophore Generation

The development of pharmacophoric of Adenine derivatives was carried out *via* Schrodinger software (LLC, New York, NY). In which, phase tool was applied to find the common pharmacophoric feature of this series compounds. Whereas, Ligprep tool was used for prepare ligands in order to get stable conformational of structures and attaches hydrogen’s which neutralize the charges at a user-defined pH. The most stable conformation was obtained *via* conversion of these structures into 3D structures.

The activity threshold was assigned 5 for the active and 4 for inactive. This activity threshold was preferred on the basis of IC_50_ (Table **2**).

pharmacophore site was used to make the common pharmacophore hypothesis of active ligands *via* a tree-based partitioning. However, the atoms of all ligands were assigned by pharmacophore H-bond acceptor (A), features aromatic ring (R), Hydrophobic group (H), and negative charge group (N), H-bond donor (D), positively charge group (P) [11].

According to pharm set of ligands, scoring pharmacophore was completed to find the best hypothesis, where the scoring algorithm reveals from the alignment of site and vectors, the number of ligands matched, selectivity, activity, volume overlap, and relative conformational energy.

### 2.3 Formulating Common Pharmacophores

The best pharmacophore hypothesis AAAP.116 was produced after the significant identification of alignment and survival scores of active ligands in Table (**3**). The survival score was 3.707. Thereby, the pharmacophore hypothesis contains following features like as three acceptors of a pink color sphere with three arrows; besides, there was one positively charged group with blue color. The 2D pharmacophore was shown on the base of atoms present in the predicted hypothesis of a pharmacophore, where the unsubstituted Nitro at the position of five-member aromatic ring and the aromatic fused ring has a Nitro near the NH_2_ group showed hydrogen bond acceptor (A1) of the one pink spheres with arrows and one positively charge group (P9) with blue color contained adenine ring. On the other hand, the sugar moiety contained OH- group and the substitution of methyl alcohol were show two hydrogen bond acceptor (A2 and A4) of the two pink spheres with arrows.

### 2.4 Building of 3D-QSAR Models

PLS (Partial Least Square) method was applied to the development of QSAR by dividing the dataset into a training set (20%) and remaining test set in randomly selected Table (**4**). In the present study, Phase was used for the generation of the QSAR model using an atom-based model [12], which is more significant to investigate the structure-activity relationship. The model was selected, where a molecule is reacted as a set of overlapping vander Waals spheres According to normal set rules, in the hydrogen bond donors (D); hydrogen attached to the polar atoms. C–H hydrogens, carbons, and halogens are the part of hydrophobic/non-polar(H); the negative ionic charge atoms are classified as negative ionic (N); non-ionic oxygen and nitrogen are the part of electron-withdrawing (W); Positive ionic charge are the positive ionic (P); Moreover, atoms are miscellaneous (X);

Besides, during the development of QSAR, Vander walls models of the aligned training set molecules were placed in a regular grid of cubes. The development of 3D-QSAR was performed and generated for the preferred hypothesis by 5 members in the training set. One component PLS factor model along with good statistics was obtained.

### 2.5 Homology and Docking Methodology

**Fig. (1):**
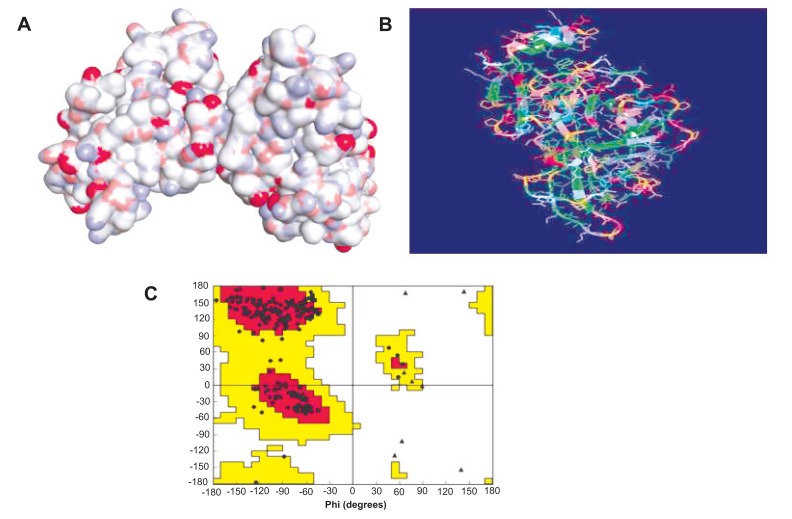
Showing the homological protein of Ebola virus: A. ionic charge of the protein. amino acid containing the dimeric structure of protein, C. Ramachandran plot of protein.

The primary structures of compounds were designed with Chem Draw Ultra 12.0 and their geometry was optimized. In another hand, Protein Data Bank (PDB) [http://www.rcsb.org/pdb/home/home.do] and National Centre for Biotechnology Information (NCBI) [https://www.ncbi.nlm.nih.gov] were used as chemical sources to obtain the reputable one homological Ebola virus sequence and further performed the Run blast and get the sequences of amino acid which is used to the homology of Ebolavirus [13]. After checking the Ramachandran plot, it was confirmed the formation of Ebola virus protein (Fig. **1**). Further, the active site was recognized with the help of CASTp database (http://sts.bioe.uic.edu/castp/). Finally, the *in-silico* molecular docking studies of the most active compound were performed using Autodock 4.1 (http://autodock.scripps.edu/) along with its LGA algorithm for computerized flexible ligand docking and binding energy identified in the form of negative Kcal/mol, probable H, and π bonds were estimated.

VP40Zaire ebola virus sequence was used for the homology of protein target *via* swiss model server [14].

>gi|973435236|gb|ALX34562.1| VP40 [Zaire ebolavirus]

MRRVILPTAPPEYMEAIYPARSNSTIARGGNSNTGFLTPESVNGDTPSNPLRPIADDTIDHASHTPGSVSSAFILEAMVNVISGPKVLMKQIPIWLPLGVADQKTYSFDSTTAAIMLASYTITHFGKATNPLVRVNRLGPGIPDHPLRLLRIGNQAFLQEFVLPPVQLPQYFTFDLTALKLITQPLPAATWTDDTPTGSNGALRPGISFHPKLRPILLPNKSGKKGNSADLTSPEKIQAIMTSLQDFKIVPIDPTKNIMGIEVPETLVHKLTGKKVTSKNGQPIIPVLLPKYIGLDPVAPGDLTMVITQDCDTCHSPASLPAVVEK

## 3 RESULT AND DISCUSSION

### 3.1 Pharmacophore Generation and 3D-QSAR Building

The main aim of present study was to investigate the 3D atoms base features and develop the potent pharmacophoric model the screening and searching potent lead towards the Ebola virus.

**Fig. (2):**
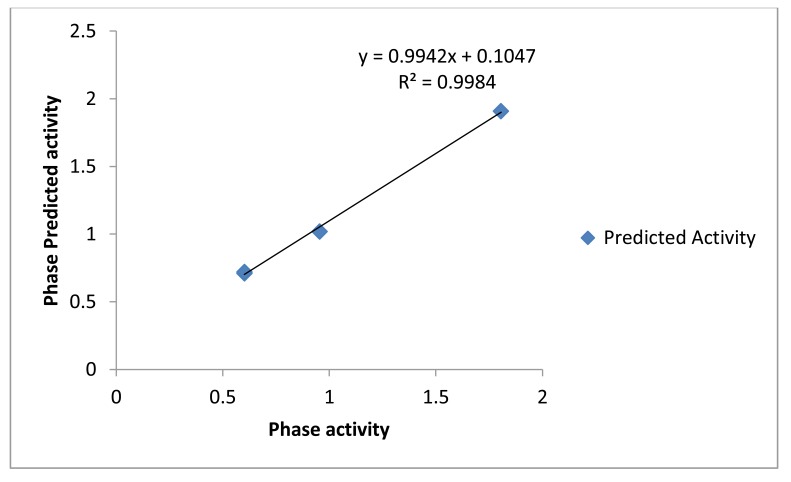
Fitness graph between experimental activity versus phase-predicted activity for training and test set compounds.

Common pharmacophore hypothesis was selected and the tree-based partition algorithm was used to generate the four variants of probable common hypotheses [15]. Further, the hypothesis of pharmacophore was selected and used to rigorous scoring faction analysis. During the development of hypotheses for pharmacophore were created for both sub-datasets. The analysis of best scores and alignment of the pharmacophore hypothesis, AAAP.116 was selected to generate the atom-based 3D-QSAR model (Table **5**), which depends on the IC_50_ activity against Ebola virus replication in Vero E6 cells and phase predicted activity (Fig. **2**).

The pharmacophoric hypothesis consisted of the three acceptors (A) and also one has the positive ionic (P) features. This hypothesis was measured on the base of regular performance of multiple runs and precious statistical analysis. On the other hand, the alignment of the pharmacophoric hypothesis with the fitness score and the better fit ligands are shown in Table (**3**). The alignment hypothesis of inactive and active scoring was shown in Fig. (**3**). Moreover, the 3D-QSAR model was generated after the choice of best hypothesis scores. The fitness of both models was shown the higher degree of robustness. One PLS factors were used to generate the best QSAR model and the LOO method was also applied to determine the coefficient value (r^2^) of 0.98 and cross-validated correlation coefficient (q^2^) of -0.058. In this regards, we were found the higher F value, which demonstrates that the statistical significance in both models and highly supported *via* lower variance ratio (p) values which intensify a greater degree of confidence. However, a small standard deviation showed the best fitness for the QSAR model. The root mean square error and Pearson’s (r^2^) display the predictive ability of the test set of both models. The significance of the productive model was obtained where all significant values of data plotted around the best fit lines. Finally, the similar trend was found in the observed and predicted value which exhibited that similar predictive values show the better prediction of the model.

### 3.2 QSAR Visualization

3D structure characteristics of atomic cubes exhibit the color according to the coefficient values shown in Fig. (**4**). The cubes were based on the view effect of acceptor and ionic positive effect with the positive coefficient and negative coefficient, which by characterize dark blue for the positive coefficient and dark red for the negative coefficient. Moreover, the positive coefficient shows an increasing in activity, whereas a negative coefficient demonstrates the decreasing activity. The 3D-QSAR represents the coefficient of three pink cubes containing H-bond acceptor (A1, A2, and A4) for the ether linkage is essential for biological activity. A light blue color around the Nitrogen into the aromatic ring contained positively charge group (P), which is responsible for increasing the activity towards the Ebola virus. Moreover, the red color near the methyl alcohol group was shown the decreased activity.

After observing 3D-QSAR model, the data suggested that the substitution at A1, A2, A4, and P9 are responsible for enhancing the activity against the Ebola virus.

### 3.3 Docking Study

*In silico,* molecular docking was accomplished using homological Ebola virus protein targets namely VP40 (Zaire ebolavirus) using Autodoc 4.1 beside by LGA algorithm parameter for computerized flexible ligand docking [16]. The binding affinity (kcal/mol), the number of H- and π- bonds, and the number of amino acids involved in interaction were estimated during the experiment Table (**6**). The most active compound EB_5 was shown the best docking poses along with bonds and its distance with the assigned target, shown in Fig. (**5**). The docking study of the potent compounds was performed to explore the essential amino acids which are responsible for activity and also support our pharmacophoric model on the basis of atomic bond formation of the ligand with the receptor.

The most active compound of this series showed the better (-8.0 kcal/mol) binding affinity along with bonds formation with target. There were a number of aminoacid residues involved for the formation of bonds with lead. These vital amino acids may be used as potential binding pocket for further drug development towards EV.

## CONCLUSION

The observation of result concluded that the common pharmacophore model of adenine derivatives responsible for inhibitory activity in order to Ebolavirus. Thereby, common pharmacophore alignment and 3D-QSAR models were created. This provided the significant information about the 3D chemical structure feature requirements for the target related to Ebola virus. The statistical analysis assessment indicates robustness and productivity, which ensure the reliability. Whenever, the pharmacophore models display the significant optimal feature for development or researching of novel lead toward of Ebola virus. Moreover, 3D-QSAR model explored the effect substituted of the chemical feature such as A light blue color around the Nitrogen into the aromatic ring contained positively charge group (P), which is responsible for increasing the activity towards the Ebola virus. Moreover, the red color near the methyl alcohol group was shown the decreased activity. On the other hand, pharmacophore patern of titled compunds resposive for the inhibitory activity towards the Ebola virus-like as the substitution at H-bond acceptor and positively charge group (P) into the adenine ring (Nitrogen). The substitution of the hydroxyl group of the sugar moiety of H-bond acceptor region and substitution of methyl alcohol group at the sugar moiety into another H-bond acceptor region show the increasing activity. Besides, the substitution of methyl or any other group near the sugar moiety may decrease the activity. On the other hand, the molecular docking was provided the potent binding pocket into the protein structure of Ebola virus and also provided the significant information in order to ligand-protein affinity along with bond formation with specific amino acid into the target protein. Finally, the model was developed from the QSAR and pharmacophore (hypothesis AAAP.116) for the Ebola virus inhibitor might provide the essential atomic structural requirements to the researcher for development of novel potent lead for the inhibition of Ebola virus.

## Figures and Tables

**Fig. (3):**
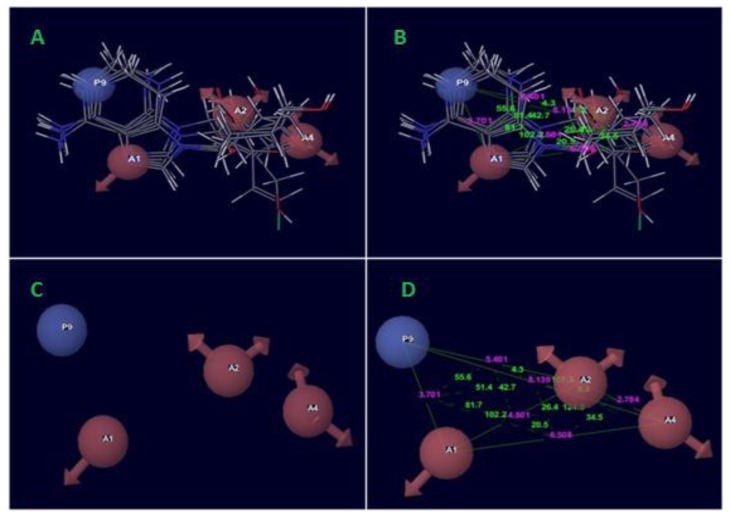
Common pharmacophore generation and validation: (a) Common pharmacophore aligned ligands three acceptor [pink sphere with three arrows] and [one positive ionic (P) (dark blue)]; (b) common pharmacophoric bond distance of ligands with pharmacophoric atoms in Å unit; (c) alignment of all active ligands to the pharmacophore; and (d) alignment of three acceptors and one positive ionic pharmacophore with distance.

**Fig. (4):**
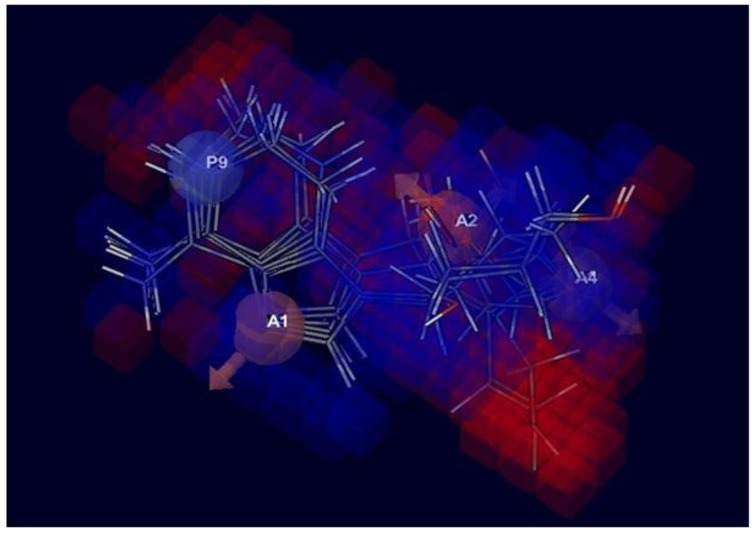
3D-QSAR visualization of various substituents’ effect: Blue cubes showing positive potential beside red cubes showing negative potential of particular substitution.

**Fig. (5):**
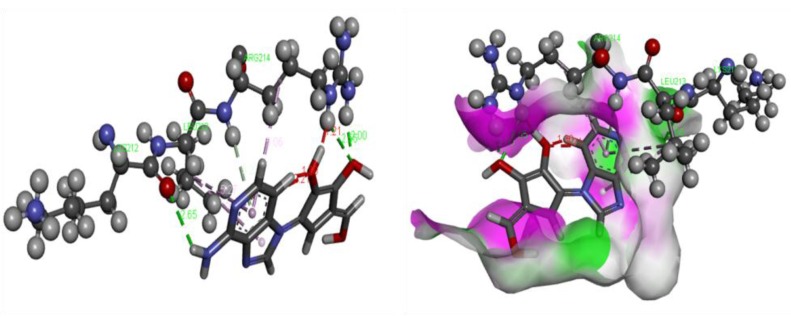
Shown the best docking binding affinity of most active compounds with VP40 (Zaire ebolavirus).

**Table 1:** Show the chemical formulae and IC_50_ value towards Ebola virus.

Sr. No.	Structures	IC_50_	Log IC_50_
**EB_1**	> 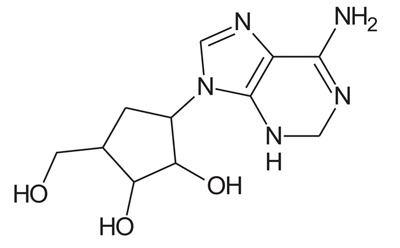	4	0.602
**EB_2**	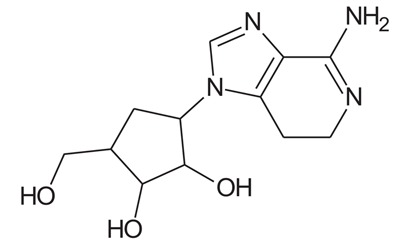	30	1.477
**EB_3**	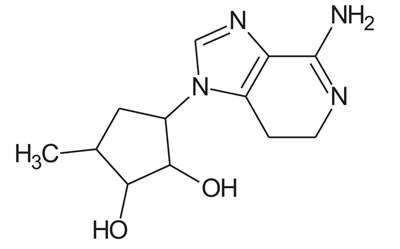	64	1.806
**EB_4**	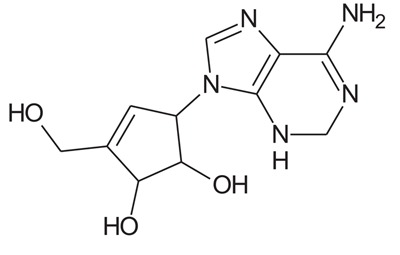	4	0.602
**EB_5**	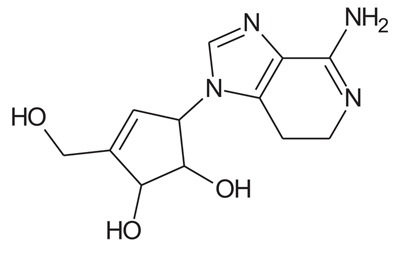	2	0.301
**EB_6**	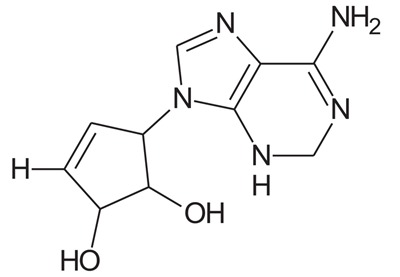	17	1.230
**EB_7**	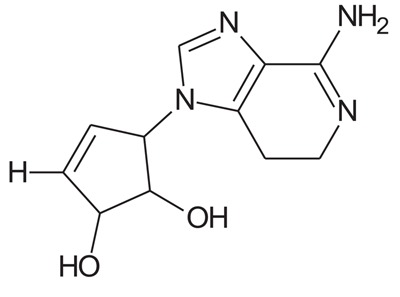	9	0.954
**EB_8**	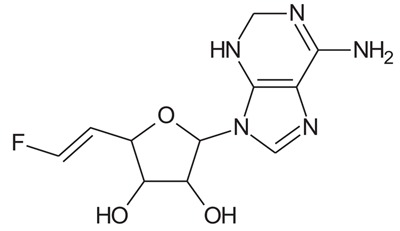	54	1.732
**EB_9**	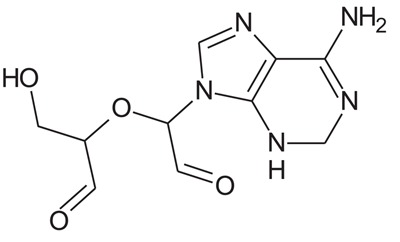	125	2.096

**Table 2:** Shown the activity threshold of 1,3,4-thiadiazole derivatives.

**Ligand Name**	**Activity**	**Pharm Set**	**Fitness**	**Sites Matched**	**Relative Energy**
**EB_1**	0.602	active	2.64	4	2.741
**EB_2**	1.477	active	2.6	4	4.514
**EB_3**	1.806	active	2.19	4	0.811
**EB_4**	0.602	active	2.92	4	1.024
**EB_5**	0.301	active	2.89	4	1.213
**EB_6**	1.23	active	3	4	1.024
**EB_7**	0.954	active	2.94	4	1.152
**EB_8**	1.732	active	2.24	4	6.249
**EB_9**	2.096	active	2.04	4	2.508

**Table 3:** Score of different parameters of the pharmacophoric hypothesis.

**ID**	**Survival**	**Site**	**Vector**	**Volume**	**Selectivity**	**Matches**	**Energy**	**Activity**
AAPR.51	3.557	0.78	0.961	0.816	1.608	9	1.018	1.23
AAPR.38	3.557	0.78	0.961	0.816	1.608	9	1.018	1.23
AAAP.116	3.512	0.75	0.958	0.804	1.481	9	1.018	1.23
AAAP.142	3.512	0.75	0.958	0.804	1.481	9	1.018	1.23
AAPR.6	3.498	0.77	0.942	0.791	1.589	9	1.045	1.23
AAPR.16	3.498	0.77	0.942	0.791	1.589	9	1.045	1.23
AAAP.67	3.491	0.77	0.94	0.782	1.466	9	1.045	1.23
AAAP.77	3.491	0.77	0.94	0.782	1.466	9	1.045	1.23
AAAP.79	3.489	0.77	0.935	0.782	1.467	9	1.25	0.954
AAAP.69	3.489	0.77	0.935	0.782	1.467	9	1.25	0.954
AADP.107	3.418	0.68	0.941	0.795	1.372	9	1.212	1.23
ADPR.56	3.414	0.69	0.936	0.784	1.523	9	1.212	1.23
AADP.98	3.414	0.75	0.894	0.765	1.366	9	1.277	0.954
AADP.96	3.411	0.72	0.9	0.787	1.337	9	1.195	1.23
AAAP.146	3.41	0.69	0.945	0.779	1.481	9	0.444	1.806
AAAP.120	3.41	0.69	0.945	0.779	1.481	9	0.444	1.806
AADP.129	3.41	0.7	0.927	0.785	1.427	9	1.518	1.23
AADP.59	3.41	0.75	0.898	0.761	1.448	9	1.25	0.954
ADPR.32	3.408	0.74	0.9	0.766	1.638	9	1.25	0.954
ADPR.46	3.407	0.74	0.899	0.769	1.526	9	1.277	0.954
ADPR.76	3.406	0.73	0.882	0.792	1.665	9	1.018	1.23
ADPR.44	3.403	0.73	0.899	0.771	1.527	9	1.045	1.23
AADP.108	3.403	0.71	0.907	0.781	1.354	9	1.418	0.602
ADPR.71	3.398	0.7	0.922	0.772	1.609	9	1.018	1.23
AADP.41	3.396	0.7	0.902	0.792	1.439	9	1.045	0.301
AADP.133	3.392	0.68	0.927	0.783	1.431	9	1.018	0.954
AADP.57	3.383	0.75	0.895	0.737	1.448	9	0.961	0.301
ADPR.5	3.374	0.71	0.893	0.767	1.529	9	1.443	1.23
ADPR.4	3.368	0.73	0.896	0.743	1.527	9	0.825	0.602
AAAP.81	3.355	0.66	0.927	0.765	1.481	9	0.811	0.602
AAAP.71	3.355	0.66	0.927	0.765	1.481	9	0.811	0.602
AAPR.20	3.34	0.67	0.91	0.758	1.605	9	0.811	0.602
ADPR.24	3.326	0.67	0.922	0.732	1.54	9	1.518	0.954
ADPR.20	3.325	0.65	0.904	0.775	1.612	9	1.018	0.301
AADP.51	3.319	0.66	0.923	0.733	1.388	9	1.045	0.954
AAPR.7	3.223	0.63	0.937	0.659	1.587	9	4.668	0.602
AAPR.17	3.223	0.63	0.937	0.659	1.587	9	4.668	0.602
AAPR.33	3.174	0.63	0.86	0.685	1.593	9	3.576	1.477
AAPR.30	3.174	0.63	0.86	0.685	1.593	9	3.576	1.477
AAPR.12	3.082	0.66	0.823	0.595	1.592	9	5.757	1.732

**Table 4:** Fitness, training set and activity data of predicted compounds.

**Ligand Name**	**QSAR Set**	**Activity**	**PLS factors**	**Predicted Activity**	**Pharm Set**	**Fitness**
**EB_1**	training	0.602	1	0.71	active	2.64
**EB_2**	training	1.477	1	1.08	active	2.6
**EB_3**	training	1.806	1	1.91	active	2.19
**EB_4**	training	0.602	1	0.72	active	2.92
**EB_5**	test	0.301	1	0.93	active	2.89
**EB_6**	test	1.23	1	0.84	active	3
**EB_7**	training	0.954	1	1.02	active	2.94
**EB_8**	test	1.732	1	1.18	active	2.24
**EB_9**	test	2.096	1	1.09	active	2.04

**Table 5:** PLS statistical parameters and 3D-QSAR models.

**ID**	**PLS Factors**	**SD**	**R-squared**	**F**	**P**	**Stability**	**RMSE**	**Q-squared**	**Pearson-R**
AAPR.51	1	0.2602	0.8245	14.1	0.03302	-0.3794	0.6842	-0.0307	0.6322
AAPR.38	1	0.2602	0.8245	14.1	0.03302	-0.3794	0.6842	-0.0307	0.6322
AAAP.116	1	0.2252	0.8686	19.8	0.02108	-0.2082	0.6933	-0.0583	0.5413
AAAP.142	1	0.2252	0.8686	19.8	0.02108	-0.2082	0.6933	-0.0583	0.5413
AAPR.6	1	0.1374	0.9511	58.3	0.004661	-1.3301	0.788	-0.367	-0.0364
AAPR.16	1	0.1374	0.9511	58.3	0.004661	-1.3301	0.788	-0.367	-0.0364
AAAP.67	1	0.1497	0.9419	48.6	0.006051	-1.4557	0.8007	-0.4116	-0.1417
AAAP.77	1	0.1497	0.9419	48.6	0.006051	-1.4557	0.8007	-0.4116	-0.1417
AAAP.79	1	0.1186	0.9635	79.3	0.002989	-1.2491	0.7529	-0.2481	0.4172
AAAP.69	1	0.1186	0.9635	79.3	0.002989	-1.2491	0.7529	-0.2481	0.4172
AADP.107	1	0.1627	0.9313	40.7	0.0078	-0.2299	0.8464	-0.5772	0.1245
ADPR.56	1	0.2025	0.8937	25.2	0.01521	-0.3227	0.8367	-0.5415	0.2858
AADP.98	1	0.1888	0.9076	29.5	0.01227	-0.188	0.7304	-0.1746	0.5767
AADP.96	1	0.257	0.8288	14.5	0.03178	-0.5437	0.5817	0.2551	0.6967
AAAP.146	1	0.1588	0.9346	42.9	0.007243	-0.5025	0.7252	-0.1579	0.5628
AAAP.120	1	0.1588	0.9346	42.9	0.007243	-0.5025	0.7252	-0.1579	0.5628
AADP.129	1	0.1641	0.9301	39.9	0.008006	0.8099	0.7937	-0.3871	0.4093
AADP.59	1	0.2485	0.8399	15.7	0.02863	-0.6287	0.6869	-0.0387	0.5404
ADPR.32	1	0.2487	0.8396	15.7	0.0287	-0.5436	0.7129	-0.1189	0.4502
ADPR.46	1	0.1893	0.9071	29.3	0.01237	-0.1546	0.725	-0.1572	0.65
ADPR.76	1	0.0632	0.9897	287.1	0.000448	0.4058	0.6192	0.1558	0.8282
ADPR.44	1	0.2026	0.8936	25.2	0.01523	-0.2396	0.7572	-0.2625	0.2791
AADP.108	1	0.1186	0.9635	79.2	0.002991	-0.6686	0.7547	-0.254	0.2485
ADPR.71	1	0.1695	0.9255	37.3	0.008824	0.8089	0.7692	-0.3026	0.5387
AADP.41	1	0.3284	0.7203	7.7	0.069	0.33	0.8206	-0.4824	0.6353
AADP.133	1	0.1642	0.9301	39.9	0.008014	0.8164	0.8018	-0.4153	0.3439
AADP.57	1	0.2495	0.8386	15.6	0.02899	-0.9388	0.6869	-0.0387	0.5562
ADPR.5	1	0.1735	0.9219	35.4	0.009484	-0.4671	0.7597	-0.2707	0.3427
ADPR.4	1	0.2209	0.8735	20.7	0.01987	-0.3611	0.7457	-0.2243	0.3231
AAAP.81	1	0.1374	0.9511	58.3	0.004665	-0.0653	0.6862	-0.0367	0.729
AAAP.71	1	0.1374	0.9511	58.3	0.004665	-0.0653	0.6862	-0.0367	0.729
AAPR.20	1	0.1739	0.9216	35.2	0.009554	-0.1645	0.7131	-0.1195	0.5763
ADPR.24	1	0.1264	0.9586	69.4	0.003626	-1.7048	0.717	-0.1317	0.5283
ADPR.20	1	0.341	0.6986	7	0.07786	0.3837	0.8475	-0.5812	0.6044
AADP.51	1	0.093	0.9776	130.7	0.001437	-1.6832	0.7072	-0.101	0.6132
AAPR.7	1	0.3438	0.6935	6.8	0.08001	0.0111	0.634	0.115	0.7229
AAPR.17	1	0.3438	0.6935	6.8	0.08001	0.0111	0.634	0.115	0.7229
AAPR.33	1	0.375	0.6353	5.2	0.1063	-0.0898	0.6637	0.0301	0.599
AAPR.30	1	0.375	0.6353	5.2	0.1063	-0.0898	0.6637	0.0301	0.599
AAPR.12	1	0.1439	0.9463	52.9	0.005366	0.6401	0.7928	-0.3837	-0.2362

**Table 6:** Docking affinity of titled compound with assigned VP40 (Zaire ebolavirus).

**Ligands**	**Receptors**	**Binding Affinity [kcal/mol]**	**Amino Acids Involved in Interaction**	**H-bonds**		**π-bonds**
5	Ebola virus	-8.0	PRO A 97 LEU A 98 GLY A 99 ARG A 148 GLN A 155 PHE A 157 LEU A 158 PHE A 161 LYS A 212 LEU A 213 ARG A 214 LEU A 288 PRO A 290 CYS A 314 HIS A 315 SER A 316	2	4	
